# Non-Anticoagulation Strategies Aimed at Primary Stroke Prevention in Nascent Atrial Fibrillation

**DOI:** 10.3390/biomedicines13030660

**Published:** 2025-03-07

**Authors:** Luca Sgarra, Vanessa Desantis, Andrea Matteucci, Vincenzo Paolo Caccavo, Federica Troisi, Antonio Di Monaco, Francesco Mangini, Grigorios Katsouras, Andrea Igoren Guaricci, Michele Luca Dadamo, Fabrizio Fortunato, Carmela Nacci, Maria Assunta Potenza, Monica Montagnani, Massimo Grimaldi

**Affiliations:** 1 Cardiology Unit, Medicine Department, General Hospital “F. Miulli” Acquaviva delle Fonti, 70021 Bari, Italy; 2 Pharmacology Section, Department of Precision and Regenerative Medicine and Ionian Area (DiMePRe-J), University of Bari Aldo Moro Medical School, 70124 Bari, Italymonica.montagnani@uniba.it (M.M.); 3 Clinical and Rehabilitation Cardiology Unit, Emergency Department, San Filippo Neri Hospital, ASL Rome 1, 00135 Rome, Italy; 4 Department of Experimental Medicine, University of Rome Tor Vergata, 00133 Rome, Italy; 5 Cardiology Unit, Department of Precision and Regenerative Medicine and Ionian Area (DiMePRe-J), University of Bari Aldo Moro Medical School, 70124 Bari, Italy; 6 Department of Cardiology, Azienda Ospedaliera Universitaria Policlinico Paolo Giaccone, 90127 Palermo, Italy

**Keywords:** stroke, atrial fibrillation, preventive therapy

## Abstract

At its earliest appearance, atrial fibrillation (AF) is often unnoticed, asymptomatic, and/or merely device-detected. Widespread use of heart-rate monitoring technologies has facilitated such “nascent atrial fibrillation (nAF)” recognition. Consequently, clinicians face a growing number of patients affected by new-onset AF in the absence of a definite indication for anticoagulation due to several counterarguments: (1) a CHA_2_DS_2_-VA score ≤ 1 in otherwise apparently healthy subjects; (2) an uncertain embolic/hemorrhagic benefit/risk ratio with anticoagulation; (3) EKG demonstration and confirmation of AF; and (4) existence of a pathogenic mechanism other than atrial hypercoagulability. In this frustrating limitation of pharmacological options, cardiologists may miss a complete comprehension of drugs with proven anti-ictal potential, whose administration may serve both as a bridge strategy toward future anticoagulation and as a consolidative strategy paralleling anticoagulation. This review aims to summarize and elucidate such therapeutic strategies and their preventative mechanisms.

## 1. Introduction

Subclinical AF refers to those very short AF episodes detected by devices needing EKG confirmation and unnoticed by the patient. On the other hand, paroxysmal AF is diagnosed by EKG, is often symptomatic, and spontaneously terminates within 7 days [1].

AF may be symptomatic or asymptomatic. AF is classified based on the temporal characteristics of the arrhythmia. When AF is first detected, it is designated as “recent-onset” (present for <48 h). AF is considered recurrent when a patient develops ≥ 2 episodes. Episodes that terminate spontaneously (by consensus within 7 days) are classified as paroxysmal, while those lasting > 1 week or requiring cardioversion (electrical or pharmacological) are considered persistent. The successful termination of AF does not alter the classification of persistent AF. Once cardioversion is not successful, or when cardioversion is not pursued according to a clinical choice, AF is classified as permanent.

Recent studies have concentrated on the frequency of asymptomatic, relatively short pseudo-AF episodes, which have been termed atrial high-rate episodes (AHREs). An AHRE is easily detected in pacemaker or implantable cardioverter defibrillator (ICD) recipients, and subsequently, it anticipates a manifestation of AF so that it occurs in the absence of AF diagnosed by the usual methods of electrocardiography or Holter monitoring. The definition of AHREs refers to episodes with an atrial lead rate ≥ 170 bpm and lasting > 5–6 s, needing visual inspection predominately to reduce the inclusion of electrical artifacts. A definition of 5 min has been found based on previously published data [2,3,4,5,6]. Similar episodes may also be detected by wearable external cardiac monitors and/or implantable loop recorders, but they are not defined as AHREs, being encompassed under the broad term of subclinical AF. Arrhythmias that have EKG characteristics of AF and last long enough to alert for a 12-lead EKG recording should be considered clinical AF.

AHRE and subclinical AF are sometimes erroneously considered interchangeable terms, with the latter including the former. Subclinical AF refers to any asymptomatic AF detected on continuous monitoring devices. Importantly, the use of the terminology “subclinical AF” requires the absence of symptoms and previously documented AF episodes. In between these two entities, there is symptomatic AF lasting very few minutes, with the latter being currently nameless. All these entities here will be referred with the term “nascent AF” (nAF).

Since nAF onset mostly affects otherwise healthy subjects or those with mild disease, such patients may be unsuitable for anticoagulation mainly because (1) they do not have a CHA_2_DS_2_-VASc score > 1, (2) their anticoagulation-related hemorrhagic risk may exceed the benefit of embolic protection, and (3) anticoagulation prescription requires EKG demonstration of true AF, and such processes might reveal very challenging outcomes for the reasons outlined above.

Nonetheless, subclinical AF recognition leads to an early 2.5-fold independent increase in stroke risk (HR, 2.50; 95% CI, 1.28 to 4.89; *p* = 0.008) [6]; this finding aligns with other studies [7] and meta-analytic evidence [8], further reinforcing the necessity for proactive diagnosis and tailored therapeutic strategies. As mentioned, the benefit/risk ratio of anticoagulation strategies in nAF has been proven unfavorable with respect to embolic/hemorrhagic risk ratio reduction [9]. Interestingly, even though anticoagulation is most beneficial in paroxysmal AF, the disparity between AF episodes and stroke onset suggests the existence of concomitant underlying pathophysiological mechanisms [7]. In this context, the term Embolic Stroke of Undetermined Source (ESUS) [10] has been introduced to define a subtype of ischemic stroke that is thought to be caused by an embolism but without an identifiable source/trigger, including nAF.

The recent concept of “atrial cardiomyopathy” (AC) as an anatomical–electrophysiological entity serves to explain the processes involved in ESUS strokes (which have been previously regarded as cryptogenic) as well as in interatrial septum defects (i.e., Patent Foramen Ovale, PFO) and thrombophilia [11,12], although the latter does not seem to fit the concept as well. However, AC is mainly a postulated concept, substantially lacking any robust pathogenic background and limiting itself to a “higher than normal amount of atrial fibrosis” very often predicated by nAF. In light of this uncertainty, cardiologists often encounter the challenge of treating apparently healthy patients and may benefit from shifting pharmacological strategies toward more focused AF-onset and stroke-preventive strategies other than anticoagulation. These strategies include the use of statins to reduce inflammation and stabilize atherosclerotic plaques, antihypertensive medications such as ACE inhibitors and ARBs to improve vascular health, and agents like colchicine to modulate inflammation. Additionally, emerging evidence suggests that GLP1-RA and certain antidiabetic drugs may offer cerebrovascular protection, making them potential options for stroke prevention and AF worsening in select populations.

## 2. Stroke Prevention Drugs

Stroke prevention strategies encompass different classes of drugs in primary prevention. There are various mechanisms involved that focus on (a) anti-inflammatory effects, (b) glucose-controlling activities, (c) LDL/triglyceride-lowering effects, (d) anti-hypertensive- and angiotensin-related homeostasis control, (e) folate cycle-related metabolic balance, (f) Obstructive Sleep Apnea Syndrome (OSAS)-related treatments, and (g) miscellanea.

### 2.1. Statins

In a number of trials investigating lipid-lowering strategies to reduce CV risk, although not the primary endpoint, stroke is a secondary or composite endpoint [13,14]. Current guidelines suggest the use of lipid-lowering treatments for primary prevention of both cardiovascular diseases and stroke [15,16]. This is consistent with the observation that a 1 mmol/L decrease in LDL cholesterol levels is associated with a decreased incidence of stroke [17]. Statins are widely recognized for their role in reducing the risk of ischemic stroke by lowering LDL cholesterol and stabilizing atherosclerotic plaques [18]. Similarly, it is well accepted that statins exert cardiovascular protective effects that are independent of LDL cholesterol lowering (called pleiotropic effects), such as those resulting from the inhibition of the production of prenylated proteins (including farnesyl pyrophosphate and geranylgeranyl pyrophosphate) in the cholesterol biosynthetic pathway. These additional effects comprise reduced vascular inflammation, improved endothelial function, decreased platelet aggregation, and enhanced fibrinolysis, reducing the overall risk of thromboembolic events and exerting neuroprotection [19,20,21].

The canonical lipid-lowering effect of statins depends on their ability to competitively inhibit the 3-hydroxy-3-methylglutaryl coenzyme A (HMG CoA) reductase, the rate-limiting step in the hepatic biosynthesis of cholesterol. The reduced intracellular concentrations of cholesterol stimulate the migration of sterol-regulating element-binding proteins (SREBPs) to the nucleus, where transcription of LDL receptors is activated. Finally, the translocation of LDL receptors to the cell membrane favors the cholesterol uptake from the bloodstream, helping to prevent atherosclerotic plaque development and progression [22].

Concomitantly, statins reduce circulating levels of inflammatory markers such as C-reactive protein (CRP), leading to a prognostic benefit both in apparently healthy patients [23] and in patients with acute coronary syndrome (ACS) [24]. Moreover, statins are able to decrease circulating levels of interleukin-6 (IL-6) [25]. These effects are mediated through the inhibition of the nuclear factor-kappa B (NF-κB) pathway and the suppression of the NLRP3 inflammasome [26,27] mainly in monocytes [28] and endothelial cells (ECs) [29]. NLRP3 activation has been indicated at the crossroad between inflammation and cholesterol dysmetabolism since cholesterol crystals [30] and ox-LDL [31] activate TLR4, ultimately promoting NLRP3 inflammasome aggregation. Interestingly, an increased NLP3 activity has been observed in patients with AF; conversely, a significant atrial electric vulnerability promoting AF is noted in animal models of constitutively activated NLP3 [32]. On a similar note, the upregulation of NLRP3 components has been detected in cardiomyocytes and atrial tissue homogenates from post-operative AF patients [33].

TLRs are a superfamily of—mainly—transmembrane receptors with a highly conserved homology with IL1-R. Their activation by inflammatory cytokines, anti-microbial peptides, adhesion molecules, and acute-phase molecules promotes innate immunity [34]. TLRs recognize ox-LDL and molecules closely linked to inflammation, as well as hyaluronan fragments, fibrinogen, heparan sulfate, HSPs, and HMGB1. Statins suppress TLR4 expression, thus inhibiting the TLR4/NF-κB/NLRP3 cascade [35,36] and ERK5 activation [37]. Despite this wide range of anti-inflammatory activities, a potential pro-inflammatory effect of statins via caspase-1 activation and inflammasome formation has been observed [38,39,40]. These controversial findings underline the importance of starting statin therapy in patients in which inflammation represents a clinical issue and a potential trigger to nascent AF [41].

Control of oxidative stress represents another fundamental goal for stroke and atherosclerosis prevention. Oxidative stress is characterized by an imbalance between reactive oxygen species (ROS) production and antioxidant defenses. As mentioned above, a key pleiotropic effect of statins consists of the prevention of isoprenoid intermediates, farnesyl pyrophosphate (FPP), and geranylgeranyl pyrophosphate (GGPP) [42], with subsequent RAS and Rho inhibition [43]. One of the most important consequences consists of the attenuation of NADPH oxidase activities [44,45,46] following a Rac1 (a Rho protein)-mediated inhibition of membrane translocation [47]. By reducing NADPH oxidase activity, statins lower ROS production [48] and this may contribute to their beneficial effects in terms of neuroprotection under conditions that range from insulin resistance to overt CV diseases [49]. Indeed, a controlled superoxide generation may significantly reduce cerebral infarction volume [50].

The close link between oxidative stress and nitric oxide (NO) production explains the preserved bioavailability of NO under attenuated ROS generation. However, several other mechanisms have been demonstrated for the statin-mediated protection of endothelial function [51]. One of the most significant mechanisms consists of both the stabilization of endothelial NO synthase (eNOS) under stress conditions—as well as hypoxia—and an increased expression via miRNA 221/22 reduction [52,53,54]. These effects depend on the inhibition of the above-mentioned RhoA and its downstream effector Rho-associated kinase (ROCK) [55]. Inhibition of the Rho/ROCK pathway leads to PI3K/Akt pathway activation and subsequent eNOS phosphorylation [56]. Interestingly, at low doses, statins increase the expression of eNOS and promote angiogenesis [57], whereas at high doses, statins seem to limit angiogenesis and the FPP-GGPP/RAS-Rho pathway, ultimately reducing EC proliferation and migration [57]. Noteworthy, such biphasic pleiotropic action [58] is a double-edged cutting mechanism since high doses can limit plaque growth but might concomitantly enhance the risk of bleeding.

The Justification for the Use of Statins in Prevention: An Intervention Trial Evaluating Rosuvastatin (JUPITER) trial aimed to assess potential benefits with respect to stroke occurrence in patients exhibiting a C-reactive protein (CRP) increase (>2 mg/L) and no hypercholesterolemia. While rosuvastatin assumption significantly decreased early stroke incidence, a slightly higher incidence of diabetes was observed during a 4-year follow-up period [59]. Concerns related to a potential glucose intolerance under a statin assumption [60] might be explained by the statin-mediated activation of NLRP3 inflammatory vesicles [61]. Nevertheless, current opinions favor the substantial benefits of statins in reducing CV complications, which outweigh the risks associated with their side effects [62]. Although the benefit/risk ratio of statin assumption should be cautiously evaluated in patients with nascent AF and increased levels of CRP, literature evidence should be carefully analyzed as well. Indeed, while prevention of one hard endpoint (composite of myocardial infarction (MI), stroke, and death) was observed during a 4-year follow-up for every 39 patients treated with statins [63,64], the incidence of one case of diabetes was observed for every 255 patients treated with statins for 4 years [60].

The Stroke Prevention by Aggressive Reduction in Cholesterol Levels (SPARCL) trial examined the effects of high-intensity statins (atorvastatin 80 mg/day) in patients already affected by stroke/TIA and low-to-moderate increases in LDLc. Besides a significant reduction in ischemic stroke recurrence, the SPARCL trial reported a small increase in hemorrhagic stroke in the statin group [65]. Although consistent with findings of a previous meta-analysis [66], a more recent meta-analysis of 23 RCTs and 19 observational studies including 248391 patients did not show evidence of any association between hemorrhagic stroke and statin assumption [67]. Overall, it looks reasonable that a slight risk of addiction in hemorrhagic stroke is outweighed by the considerable protection from ischemic stroke under statin treatment [68].

The results from the Heart Protection Study (HPS) [69] and from the PROspective Study of Pravastatin in the Elderly at Risk (PROSPER) [69] corroborate meta-analytic evidence [70], reporting no significant effects of statins on cognitive decline.

As briefly mentioned above, side effects of statins are usually associated with long-term treatment and high cumulative doses [71]. Particularly for the prevention of diabetes, the practice of switching to a different statin or employing a lower dose remains the most prevalent and reasonable clinical approach.

Turning to a clinical perspective, statin therapy has been shown to reduce the risk of a first stroke in adults who qualify for lipid-lowering treatment and are at higher cardiovascular risk, as evidenced by findings from multiple meta-analyses ((RR 0.78; 95% CI, 0.68–0.89) [72]; (RR 0.81; 95% CI, 0.75–0.87) [73]).

Interestingly, some meta-analytic evidence highlights that atorvastatin use is associated with a reduced burden of new-onset AF in patients at a high risk of coronary artery disease (CAD) (OR 0.55–0.38 to 0.81) [74]. Unfortunately, these findings are weakened by more than 50% heterogeneity among patients recruited, thus generating some skepticism. Regarding rosuvastatin, a meta-analysis [75] of four RCTs, primarily conducted in high-CV-risk patients, demonstrated a 30% reduction in the risk of AF onset (RR 0.70–0.54 to 0.91).

Noteworthy, ACC/AHA guidelines on stroke primary prophylaxis [16] recommend statin therapy for individuals with LDL-c levels > 190 mg/dL even in young adults (as early as 20 years old) with a I/A class of recommendation. Meanwhile, the ESC guidelines on dyslipidemia [76] suggest considering statin therapy for LDLc levels > 116 mg/dL in low-risk patients, with a IIa/A class of recommendation. Taken together, these guidelines strongly support the use of statins for primary stroke prevention.

A more detailed description of potential protective effects for specific statins is described in the following paragraphs.

#### 2.1.1. Rosuvastatin

As previously mentioned, the JUPITER trial [65] aimed to assess the potential benefit of rosuvastatin in reducing stroke occurrence among patients exhibiting a C-reactive protein (CRP) increase (>2 mg/L) but without hypercholesterolemia. Over a 1.9-year follow-up, rosuvastatin administration led to a remarkable 48% reduction in stroke risk (HR, 0.52; 95% CI, 0.34 to 0.79; *p* = 0.002). Intriguingly, a subanalysis of the JUPITER trial stated that rosuvastatin use might decrease the incidence of AF (0.73–0.56 to 0.94) [77].

Such findings were further confirmed by the Heart Outcomes Prevention Evaluation–3 (HOPE-3) subanalysis [14], which enrolled patients without manifest CV diseases (CVD) but with mild hypercholesterolemia and hypertension. This study demonstrated a 47% reduction in stroke occurrence (HR, 0.53; 95% CI, 0.37 to 0.78; *p* = 0.001).

#### 2.1.2. Simvastatin

The Heart Protection Study (HPS) [78] was a megatrial enrolling 20536 patients affected by peripheral artery disease (PAD) or diabetes and treated with 40 mg of simvastatin, regardless of baseline LDL-c, or placebo. Among its various outcomes, the study provided stroke reduction data over a 5-year-long period. In these high-CV-risk patients, simvastatin therapy resulted in a significant 35% reduction in stroke risk both in PAD and in non-PAD patients (RR 0.75–0.66 to 0.85). Based on these findings, simvastatin emerges as an attractive therapeutic option for reducing stroke incidence in patients affected by PAD/CAD and with a moderate-to-high CV risk.

### 2.2. Antidiabetic Drugs

A comprehensive analysis of 48 randomized trials evaluated the impact of eight distinct glucose-lowering medications on stroke risk. The results indicated that only GLP-1 receptor agonists (GLP-1RAs) and thiazolidinediones (TZDs) significantly reduced the likelihood of stroke [79]. In contrast, Sodium-Glucose Transporter-2 Inhibitors (SGLT2-Is), while highly effective in reducing major CV outcomes such as heart failure hospitalizations and kidney disease progression, do not appear to have a significant impact on cerebrovascular protection and stroke prevention [80].

#### 2.2.1. Thiazolidinediones (TZDs)

TZDs are PPARγ receptor agonists, a class of antidiabetic drugs exerting insulin-sensitizing properties and able to improve cardiovascular homeostasis. In the vessels, their activity is able to favor the production of endothelial NO, the inhibition of adhesion molecule expression, the suppression of the activation of monocytes/macrophages, a reduction in pro-inflammatory cytokine secretion, the promotion of the reverse trafficking of lipids, and the modulation of the proliferation, migration, and apoptosis of vascular smooth muscle cells (VSMCs) [81]. Taken together, the aforementioned mechanisms may contribute to counteracting atheroma growth and limiting stroke onset. However, some controversies exist since the same mechanisms leading to VSMC apoptosis may become detrimental in advanced phases of atherosclerosis when fibrous cap weakening might exacerbate plaque rupture and trigger vascular complication [81].

##### Pioglitazone

A post hoc analysis of the Insulin Resistance Intervention After Stroke (IRIS) trial involving patients affected by insulin resistance and stroke observed a 28% reduction (HR, 0.72; 95%CI, 0.56–0.92) in stroke recurrence over a period of 4.8 yrs with pioglitazone treatment [82]. The PROspective pioglitAzone Clinical Trial In macroVascular Events (PROactive) study [83] was a multicenter trial that aimed to understand whether pioglitazone was superior to a placebo in preventing macrovascular complications. Although often described as a negative trial due to the absence of benefit in the prespecified primary endpoint (MACE), a subanalysis of patients with a prior stroke revealed a significant 47% reduction in stroke recurrence (HR 0.53–0.34 to 0.85) under pioglitazone therapy [84]. Taken together, findings from both IRIS and PROactive studies highlight pioglitazone as an attractive option for secondary stroke prevention. However, a breakthrough finding emerged from a longitudinal retrospective Korean registry involving 128,171 patients with newly diagnosed T2DM, which reported a 31% reduction in first stroke occurrence with pioglitazone treatment (HR, 0.69; 95%0.59–0.80) [85]. Although no dedicated trial has been designed to specifically address the effects of pioglitazone on primary stroke prevention in insulin resistance/T2DM patients, it is reasonable that pioglitazone administration may be an effective strategy for primary stroke prevention in insulin-resistant/T2DM patients with nascent AF. In addition, a recent meta-analysis by Zang et al. revealed that TZD use was associated with a significant reduction in the risk of new-onset AF episodes (OR 0.77–0.65 to 0.91, *p* = 0.002) [86].

Current consensus, however, promotes pioglitazone as a third-line drug for secondary stroke prophylaxis. Additional clinical trials focusing on pre-diabetic patients—those with elevated blood sugar levels that do not yet meet the threshold for type 2 diabetes—would represent a valuable contribution to understanding the potential role of pioglitazone in stroke prevention within this population.

#### 2.2.2. GLP-1 Receptor Agonists (GLP-1RAs)

GLP-1RAs are highly promising drugs for cardio-/cerebrovascular protection. Consistent with growing evidence from meta-analyses [87], the stroke-preventive effects of GLP-1RAs support their use in individuals with type 2 diabetes (T2DM). This rationale is reflected in multiple clinical guidelines and consensus statements, including the 2020 Canadian Stroke Best Practices [87], the Spanish Society of Neurology consensus statement 2021 [88], the American Heart Association/American Stroke Association Guideline for the Prevention of Stroke in Patients with Stroke and Transient Ischemic Attack [89], and the Diabetes, Cardiorenal, and Metabolic Diseases Multispecialty Task Force [90].

These recommendations are based on recent and consistent meta-analytic efforts: a meta-analysis of results from eight trials evaluating GLP-1RAs demonstrated a significant reduction in the fatal/nonfatal stroke outcome compared to the placebo (HR 0.83–0.76 to 0.92) [91]. Similarly, a more recent meta-analysis confirmed the aforementioned results, reporting a significant decrease in combined fatal/nonfatal strokes (HR 0.83–0.76 to 0.92) (HR 0.84–0.76 to 0.93). However, no significant reduction was observed for fatal stroke alone [92].

Currently, no subanalysis on stroke incidence is available from the Semaglutide Effects on Heart Disease and Stroke in Patients with Overweight or Obesity (SELECT) trial [93], which evaluates the impact of semaglutide 2.4 mg once weekly (QW) in non-diabetic overweight or obese individuals with CVD. However, future analyses from this trial could provide valuable insights into the potential stroke-preventive effects of semaglutide in this population.

In this otherwise optimistic scenario, the potential of GLP1-RAs to protect against AF onset is still uncertain: Monami et al. [94] found that GLP1-RA administration did not influence AF onset, a finding consistent with results reported by Chan et al. [95] and a subanalysis of the REWIND trial (which compared dulaglutide vs. a placebo in patients with T2DM) [96]. Unfortunately, very recently, some concerns have emerged from a French registry suggesting a potentially harmful effect of GLP-1RAs on AF onset [97]. These findings warrant further investigation to clarify the relationship between GLP-1RA therapy and AF risk.

##### Dulaglutide

As previously mentioned, several trials investigating the MACE-protective effects of various GLP-1RA molecules have reported a stroke-preventative benefit. Among them, the Researching cardiovascular Events with a Weekly INcretin in Diabetes (REWIND) trial stands out. The REWIND trial [98] randomized a large cohort of 9901 patients affected by T2DM and either CVD (including stroke) or CV risk factors to receive weekly dulaglutide or the placebo. Over a 5.4-year follow-up, dulaglutide treatment led to a 24% reduction in stroke incidence (HR 0.76–0.62 to 0.94). Interestingly, a REWIND subanalysis assessing AF onset found no increased risk of AF with dulaglutide administration [96]. However, since the study included patients with a history of ischemic stroke, myocardial infarction, or unstable angina, its findings do not fully elucidate the potential benefits of dulaglutide in primary stroke prevention. Despite this limitation, given that GLP-1RAs are recommended as a second-line therapy for T2DM patients, dulaglutide should be regarded as the best-characterized GLP-1RAs regarding stroke prevention, with minimal concerns about AF risk [96].

##### Semaglutide

The Trial to Evaluate Cardiovascular and Other Long-term Outcomes With Semaglutide in Subjects With Type 2 Diabetes (SUSTAIN-6) primarily enrolled high-CV-risk patients, with 10% having a history of stroke and 30% having experienced myocardial infarction. The study demonstrated a 39% stroke risk reduction (HR 0.61–0.38 to 0.99) with semaglutide over 2.1 years. The concordance between findings from the REWIND and SUSTAIN-6 trials, along with positive results from meta-analytic evidence [91], led to the recent update in the AHA/ACC primary stroke prevention guidelines, which now recommend GLP-1RAs as a potential stroke-preventative therapy in T2DM patients with suboptimal glucose control (Hba1c > 7%) [16]. Interestingly, a recent meta-analysis involving 10 trials [99] that recruited obese and diabetic patients and examined semaglutide administration, regardless of oral or subcutaneous route, was associated with a striking 42% reduction in incident AF (RR 0.58–0.4 to 0.85). However, the stroke reduction effect was not statistically significant, though it showed a favorable trend (RR 0.76–0.47 to 1.24). Although these findings do not align with the significant stroke reduction observed in the SUSTAIN-6 trial, this discrepancy may be attributed to the underpowered number of stroke events in the other studies included in the meta-analysis [100].

### 2.3. Folate Cycle Fortification

Folate cycle fortification refers to those strategies basically aiming at lowering plasma homocysteine levels, which have been identified as the main biomarker of folate cycle disturbances. As a metabolic pillar of cellular homeostasis responsible for one-carbon-unit metabolism, defects in the folate cycle may activate several mechanisms contributing to stroke. Methyl surcharge may cause remethylation of alternative substrates such as one-for-all L-arginine [101]. Asymmetric dimethylated arginine (ADMA) becomes L-arginine’s strongest competitor and antagonist of endothelial NO synthase (eNOS), promoting endothelial dysfunction, oxidative stress, and a pro-inflammatory, pro-thrombotic milieu. Several trials, mainly enrolling patients with a high-CV-risk profile, have been meta-analyzed by a warranted Cochrane trial sequential and meta-analysis. The results obtained highlight a 10% stroke risk reduction with respect to the placebo (RR 0.90–0.82 to 0.99) in the interventional arm based on the administration of several B-complex vitamins (including different doses of folic acid) [102]. Although some confounding factors (such as the inclusion of healthy and ill individuals, such as stroke patients, end-stage renal disease patients, and suspected and overt coronary artery disease (CAD) patients) may result in moderate-quality evidence, the current opinion on folate fortification is leaning toward a beneficial effect on stroke primary prevention [16].

#### Folic Acid

The China Stroke Primary Prevention Trial (CSPPT) is a megatrial designed to enquire whether folic acid added to an enalapril-based therapy is more effective than enalapril alone for stroke prevention even without further lowering blood pressure in substantially healthy patients. More than 20,000 patients, with an average age of 60 years, were enrolled and followed for 4.8 years. Recruitment was terminated ahead of time due to significant benefits showing a 24% first-stroke-risk reduction (HR 0.76–0.64 to 0.91). This is so far the only trial supporting a significant anti-stroke activity of folic acid administration in younger and seemingly healthy subjects. Folic acid administration is considered the “least common denominator” in homocysteine-lowering strategies; since elevated homocysteine levels are associated with both paroxysmal and persistent AF [103], it is not surprising that folic acid administration may be regarded as a potential strategy to prevent and treat nAF.

### 2.4. Obstructive Sleep Apnea Syndrome (OSAS)

OSA is an independent risk factor for all stroke patients [104,105]. Additionally, it is accepted that OSA is a potent trigger of nocturnal AF primarily due to subsequent profound parasympathetic activation. Indeed, the Sleep Heart Study (SHS) [106] provided compelling evidence of a temporal relationship between AF and OSA, showing an impressively higher risk of arrhythmia (OR 17.5; 85% CI, 5.3–58.4) within 90 s following a respiratory disturbance compared to normal breathing. The median AF duration was 7 s, with the longest episode lasting 5 min, aligning with the nAF concept. Aside from AF-related embolic stroke, several other mechanisms have been postulated in OSA patients. Intermittent hypoxia causes intense oxidative stress due to nonprotein-bound iron increase [107,108], and free radicals contribute to vascular injury and plaque complications. Moreover, oxidative stress is accompanied and sustained by a decreased expression of eNOS and an impaired activation of phospho-eNOS [109,110]. A compensatory increase in erythropoiesis—probably the main efficient mechanism triggered by hypoxia [111]—ultimately led to a higher hematocrit value and increased blood viscosity, which are both important determinants for stroke. Interestingly, OSA has been associated with hypercoagulability via a rise in homocysteine, similar to folate cycle disturbances [112]. Of note, some preliminary evidence suggests that OSAS-limiting surgery may reduce homocysteine levels [113], offering a potential stroke-protective effect. Finally, inflammation assessed by increased CRP and interleukin-6 (IL-6) levels match OSAS severity [114,115] and may further contribute to vascular meta-inflammation processes, early atherosclerosis, arterial stiffness, and endothelial dysfunction, ultimately favoring stroke onset.

#### Continuous Positive Airway Pressure (CPAP)

CPAP is traditionally regarded as “the” OSAS therapy. However, its widespread adoption is limited by poor patient compliance and technological complexity. These factors, combined with the relative rarity of stroke events, may help explain the conflicting evidence regarding CPAP’s role in stroke prevention. While a meta-analysis of observational studies suggested that CPAP therapy can significantly reduce stroke risk (RR 0.27–0.14 to 0.53) [116,117], randomized controlled trials (RCTs) have not consistently demonstrated a significant reduction in stroke risk with CPAP therapy (OR 0.93–0.7 to 1.24) [116]. Very few RCTs have specifically evaluated primary stroke prophylaxis in otherwise healthy patients, and the few available were neither addressed to explore stroke alone nor powered for longer-term follow-ups [118,119,120]. A single RCT enrolling 725 substantially healthy patients with no prior cardio-/cerebrovascular events showed a significant reduction (IDR 0.72–0.52 to 0.98) in a time-normalized composite endpoint (including stroke) in those patients with higher CPAP compliance (>4 h/night) [119]. Nonetheless, even in the absence of conclusive evidence demonstrating a reduction in stroke risk, CPAP therapy appears to be an effective strategy for reducing the incidence of AF (OR 0.51–0.27 to 0.97) [121]. Taken together, and based on its potential benefit in stroke prevention, CPAP therapy has been favorably recommended for primary stroke prevention and has been included in the latest ACC/AHA guidelines [16].

### 2.5. Non-Canonical Anti-Inflammatory Strategies

Inflammation has recently emerged as an independent risk factor for cardio-/cerebrovascular events, with several biomarkers such as high-sensitivity CRP [122], IL-6 [123], and interleukin-1 beta (IL-1β) [124] linked to an increased likelihood of adverse cardio-cerebrovascular outcomes, irrespective of cholesterol levels.

Evidence suggests that hs-CRP serves as a prognostic marker [122], while IL-6 [123] and IL-1β [125] play causal roles in the development of atherosclerosis. CRP, in particular, exerts direct effects on endothelial cells, including the upregulation of adhesion molecules and tissue factors (TFs) [126,127,128], the stimulation of monocyte chemoattractant protein-1 (MCP-1) [129] and matrix metalloproteinases (MMPs) [130], and the increased production of inflammatory cytokines [131]. Additionally, CRP disrupts endothelial homeostasis by enhancing inducible NO synthase (iNOS) activity and superoxide production while reducing protective factors like eNOS-mediated NO release, prostacyclin, and tissue plasminogen activator (tPA) [126,132].

#### Colchicine

Colchicine, a microtubule-targeting agent, binds specifically to tubulin [133], disrupting the cytoskeleton of inflammatory cells. At low concentrations, it halts microtubule growth and cell migration and inhibits neutrophil-mediated superoxide production [134,135,136] while at higher concentrations, it promotes depolymerization and halts cell division. Thus, low doses of colchicine affect several microtubule-dependent processes such as the following: (1) Neutrophil chemotaxis [137]. This results from the persistence of the myeloid inhibitory C-type lectin-like receptor (MICL) that acts as an immune checkpoint, therefore limiting neutrophile activation and damping inflammation [138]. (2) Neutrophil–endothelial cell interaction and adhesion. This is subsequent to the altered expression of E-selectin in endothelial cells and the shedding of L-selectin in neutrophils [139,140]. Moreover, the endothelial-protective effect of colchicine may result from its ability to reduce levels of ADMA, thrombomodulin (TM), and osteoprotegerin (OPG) [141]. Finally, low doses of colchicine are able to inhibit smooth muscle cell (SMC) proliferation, migration, and secretion, thus potentially being useful to counteract plaque growth when administered in the early phase of atherosclerotic vascular disease [142,143]. (3) Phagocytosis. This is secondary to the stimulation of antigen presentation [144,145,146]. (4) Platelet activation/aggregation. Since microtubular dynamics is fundamental for platelet activation, it is plausible that, even at lower doses, colchicine might slow the platelet aggregation process. At higher doses (20 nmol/L), colchicines seem to exert more evident antiaggregant effects as a consequence of inhibition on P2Y12 and collagen glycoprotein receptors [147]. The inhibition of cofilin and LIM domain kinase-1 has been hypothesized as an additional antiaggregation mechanism of high-dose colchicine [148].

Colchicine-mediated inhibition of the NF-κB pathway is another relatively recent mechanism that has been elucidated [149]. This pathway reacts to environmental attacks and drives the cells toward their final destination (apoptosis/survival/differentiation), substantially supporting inflammatory processes and innate immune response and promoting atherosclerosis. A reduced activation of NF-κB signaling has been observed in both HeLa cells incubated with colchicine [150], as well as in patients with Familial Mediterranean Fever treated with this drug [151].

The discovery of attenuated activation of the NLRP3 inflammasome in colchicine-treated neutrophils and macrophages in the setting of gout [151,152] has prompted the hypothesis that colchicine can also mitigate cholesterol crystal-induced inflammation (via NLRP3 attenuation) within atherosclerotic plaque and display potential benefits in different cardiovascular diseases [153]. Even if such therapeutic potential has been shown with high doses of colchicine [154,155], it might be possible that a sufficient cytosolic neutrophil concentration might be achieved with continuous prophylactic administration of low-dose colchicine. Indeed, with respect to plasma concentration, colchicine accumulation in neutrophils is approximately 16 times higher [156,157]. The putative role of NLRP3 inflammasome activation on AF [32,33] has been recently confirmed [158]. Of note, colchicine is able to reduce IL-6 and CRP levels in patients with CAD [159]. This is intriguing since IL-6 and CRP are biomarkers predicting AF in CAD patients [160,161,162].

By modulating the expression of inflammatory proteins and several cellular functions, colchicine could hold the potential to regulate the atherothrombotic network, involving endothelial cells, inflammatory mediators, and platelets [139,163]. Altogether, these activities position colchicine as a promising therapeutic option for mitigating inflammation and thrombosis.

Since colchicine administration goes back a long time, data concerning its safety and efficacy are consistent and reliable. Its main gastrointestinal side effects are reversible with drug discontinuation. Interestingly, a very recent meta-analysis reported that colchicine can prevent AF regardless of the maintenance dose, cumulative daily dose, and duration of therapy. The most common side effects were diarrhea and nausea, both of which can be avoided with low doses (0.5 mg/day) and a long period of administration [164].

Even if rare under low-dose administration, myopathy/rhabdomyolysis are possible side effects with colchicine. Thus, especially in elderly subjects or in patients with renal failure and statin or CYP3A4 inhibitor therapy, colchicine treatment itself or close follow-up should be carefully considered.

With these potential limitations in mind, colchicine has been recently repositioned as an attractive and effective choice in primary and even secondary prophylaxis of stroke. Disappointingly, the Colchicine in Atrial Fibrillation to Prevent Stroke (CIAFS-1) trial (ClinicalTrials.gov ID NCT02282098), aiming at elucidating colchicine’s potential for both inflammation markers and stroke reduction in AF patients, has been discontinued with no explanatory reason described. Nowadays, colchicine administration has been tested in atherosclerotic cardiovascular disease patients, primarily affected by stable CAD, even in primary prophylaxis. Both pieces of evidence arising from RCTs (RR 0.67–0.59 to 0.77) [165] and from cohort studies (RR 0.46–0.41 to 0.52) [166] consistently report colchicine as an effective strategy for stroke risk reduction. In particular, results from the Low-Dose Colchicine for Secondary Prevention of Cardiovascular Disease (LoDoCo 2) trial [167], which enrolled patients aged from 35 to 82 years affected by stable coronary artery disease according to an Agatston calcium score > 400, revealed a significant benefit in terms of stroke reduction in patients treated with low-dose colchicine (0.5 mg/day) with respect to placebo-treated patients (HR 0.72–0.57 to 0.92). Although the mechanism of the benefit of colchicine was not investigated in this trial, colchicine has been shown to acutely lower the local production of NLRP3 inflammasome-related cytokines, IL-1β, IL-18, and IL-6, in the coronary vascular bed of patients with CAD [168]. Consistent with these findings, regular colchicine use at a dose of 0.5 mg/day has been able to modify the composition of coronary atherosclerotic lesions [169]. Interestingly, colchicine has been proven as an effective and attractive strategy to reduce both recurrences and even first-onset AF in various settings, including AF ablation and myocardial revascularization, as well as in stable CAD (0.75–0.68 to 0.83) [164]. Considering colchicine’s propensity to cause gastrointestinal side effects in up to 25% of patients, repurposing of this drug for atherosclerosis is not without its own challenges. While exploring mechanisms underlying colchicine’s ability to attenuate cardiovascular risk, the above-mentioned evidence paves the way to make colchicine an attractive strategy aiming to reduce stroke risk irrespective of anticoagulation in nAF.

### 2.6. Angiotensin-Converting Enzyme Inhibitors (ACE-Is)

The ACE is a key component of the renin–angiotensin–aldosterone system (RAAS), which is responsible for generating angiotensin II (Ang II), a molecule with significant physiological and pathological effects on the cardiovascular system [170]. Moreover, ACE activity promotes bradykinin (BK) degradation. BK causes vasodilation via the release of prostacyclin, NO, and other endothelium-derived relaxing factors. Only a small portion of ACE is present in plasma, while the majority resides in vascular tissues, where it plays a central role in vascular health and disease.

Excessive Ang II contributes to CV dysfunction through several mechanisms: (1) vascular remodeling: it stimulates the proliferation of smooth muscle cells within blood vessels [171]; (2) oxidative stress: it promotes lipid peroxidation and the production of reactive oxygen species, exacerbating cellular damage [172]; and (3) inflammation and endothelial dysfunction: by increasing the expression of pro-inflammatory genes, Ang II impairs the function of endothelial cells, favoring atherosclerosis and reduced vascular flexibility [173]. Tissue ACE, rather than circulating ACE, is increasingly recognized as a critical therapeutic target in managing cardiovascular diseases. Consequently, ACE-Is provide benefits that extend beyond lowering blood pressure. It is accepted that they prevent CV complications by limiting the excessive growth of vascular smooth muscle cells, reducing arterial stiffening, stabilizing endothelial function and plaque structure, and enhancing fibrinolysis, all of which help to reduce the likelihood of thrombotic events.

#### Ramipril

Ramipril is the most lipophilic and, subsequently, the most tissue ACE-inhibiting molecule. Ramipril has been evaluated for its neuroprotective potential against stroke in high-CV-risk patients in the context of the Heart Outcomes Prevention Evaluation (HOPE) study [174]. This trial enrolled 9297 patients with a history of CAD, PAD, or stroke and at least one classical CV risk factor. The mean age was 66 years, and very few patients reported a stroke (about 10%), while half of the patients had suffered a myocardial infarction (MI). Treatment with ramipril 10 mg during a 4.5-year follow-up period resulted in a 32% stroke risk reduction (RR 0.68–0.56 to 0.84). Interestingly, according to results from the ONgoing Telmisartan Alone and in Combination With Ramipril Global Endpoint (ONTARGET) trial [175], in patients intolerant to ACE-Is and with comparable high-CV-risk profiles (except for a higher incidence of stroke, approximating 20%), 80 mg of telmisartan (the most lipophilic ARB) was non-inferior with respect to ramipril (RR 0.91–0.79 to 1.05) [176]. The very same patients enrolled in the HOPE trial have been studied to understand whether or not ramipril therapy would be able to prevent new-onset AF. Ramipril and ACE-Is failed to avoid AF occurrence in patients unaffected by left ventricular dysfunction (LVD) [177] whereas patients affected by LVD and hypertrophy [178] benefit from ACE-I treatment. Nevertheless, analysis in this subset of patients is beyond the aim of this review.

### 2.7. Angiotensin II Receptor Blockers (ARBs)

With respect to ACE-Is, which reduce Ang II levels globally and exert additional effects via bradykinin (BK) accumulation, ARBs block the AT1 receptors and enhance AT2 receptor-mediated signaling, leading to direct neuroprotective and anti-inflammatory effects [179,180]. This distinction might give ARBs unique advantages in cerebrovascular protection, particularly in inflammation-driven pathways implicated in stroke [181,182].

#### 2.7.1. Candesartan

Candesartan is largely employed in high-CV-risk settings due to its recognized ability to enhance survival, avoid hospitalization, and prevent stroke mainly in heart failure (HF) patients according to the Candesartan in Heart failure Assessment of Reduction in Mortality and morbidity (CHARM) trial [183]. Nevertheless, in the context of the Study on COgnition and Prognosis in the Elderly (SCOPE) trial [184], candesartan has been evaluated for stroke prevention in non-HF, elderly (70 to 89 yrs old) hypertensive patients with negligible percentages of previous stroke and MI in their baseline characteristics (about 4% each). The authors reported a 42% stroke risk reduction (RR 0.58–0.33 to 1.00) despite some difference in blood pressure lessening, suggesting a pleiotropic drug effect. Interestingly, similarly to ramipril, candesartan appears to be effective in preventing AF onset in HF patients [185], whereas its protection against AF onset was not observed in small patient cohorts [186,187].

#### 2.7.2. Losartan

Losartan is unique among ARBs since it displays the additional ability to stimulate BK2 receptors [188], therefore implying a promising neuroprotection potential [189]. In this perspective, the Losartan Intervention For Endpoint reduction in hypertension (LIFE) trial [190] investigated losartan anti-ictal effects in moderately hypertensive patients with cardiopathy (EKG assessed left ventricular hypertrophy), among which approximately 20% suffered from PAD/CAD, and only a tiny percentage reported a previous stroke/TIA episode (about 4%). With respect to atenolol, losartan treatment resulted in a 25% stroke reduction (0.75–0.63 to 0.89). Interestingly, a retrospective analysis of these patients (n = 9983), followed along a timeline of 4.8 yrs, stated that losartan administration is associated with a lesser extent of new-onset AF with respect to patients under treatment with atenolol (adjusted HR 0.67–0.55 to 0.83) [191], thus representing a significant variation across the ACE-I/ARB drug classes.

### 2.8. Omega-3 PUFA

Although it is timidly proposed that omega-3 polyunsaturated fatty acid (PUFA) supplementation, especially with eicosapentaenoic (EPA) and docosahexaenoic acid (DHA), confers mild protection against coronary heart disease and coronary death, the effect on stroke outcome remains uncertain [192]. Nonetheless, it is established that the higher the level of plasmatic omega-3 PUFA, the lower the risk of ischemic stroke [193]. Along the same lines, higher values of nonfasting tryglicerides correlate with a higher risk of stroke [194] and omega-3 PUFA supplementation is helpful to decrease hypertriglyceridemia. In this still unclear field, recent results from the Reduction in Ischemic Stroke With Icosapent Ethyl (REDUCE-IT) trial seem to shed some light, demonstrating a significant 25% stroke reduction in patients undergoing treatment with a highly purified omega-3 fatty acid [195].

#### Icosapent Ethyl

The goal of the REDUCE-IT trial was to assess the safety and benefit of icosapent ethyl (IPE), a prescription omega-3 fatty acid, compared with a placebo in reducing CV events among patients with high triglycerides (TGs), established CV disease, or diabetes and at least another classical CV risk factor. Overall, the results for IPE vs. the placebo on the primary CV outcome of CV death, nonfatal MI, stroke, coronary revascularization, or unstable angina was 17.2% vs. 22.0% (HR 0.75, 95% CI 0.68–0.83; *p* < 0.0001). Stroke was similarly reduced by 28% (0.72–0.55 to 0.93), according to a 2021 preliminary study [195]. However, consistent with other studies [196] and revisional processes [197], the onset of both AF or flutter was significantly higher (3.1% vs. 2.1%, P 0 0.004) in patients treated with IPE. Of note, the use of mineral oil as a placebo could have likely contributed to the LDL-C and CRP increase in the control group, potentially justifying an overestimation of benefits in IPE-treated patients [198]. In addition, no specific information was available in terms of stroke events, making it difficult to discern whether IPE administration was effective in primary rather than in secondary prevention. Based on these unclear points, the authors concluded that treatment with IPE was not recommended in the primary prevention of stroke in nAF, confining its possible use to decisions made on a case-by-case basis according to the recent AHA/ACC primary stroke prevention guidelines [16].

## 3. Discussion

In daily clinical practice, cardiologists often find the prescription of oral anticoagulation therapy challenging due to several key considerations: (1) a CHA_2_DS_2_-VASc ≤ 1 in otherwise apparently healthy subjects; (2) an uncertain embolic/hemorrhagic risk/benefit ratio with anticoagulation, where, in particular, this dilemma may occur because of a historical hemorrhagic spontaneous event; (3) difficulty obtaining EKG demonstration and confirmation of AF even when atrial high-rate episodes (AHREs) are intercepted in CIED recipient; and (4) the existence of pathogenic mechanisms beyond atrial hypercoagulability, which may contribute to stroke risk. Given these challenges, cardiologists should broaden their focus beyond atrial hypercoagulability, aiming to develop a comprehensive stroke-protective strategy that also complements anticoagulation, when necessary, by considering other pathogenic mechanisms contributing to stroke risk. Several RCTs can address this topic, with some evaluating ischemic stroke per se, as a valuable primary outcome, while others have considered it as a secondary outcome, via MACE deconstruction.

**Figure 1:**
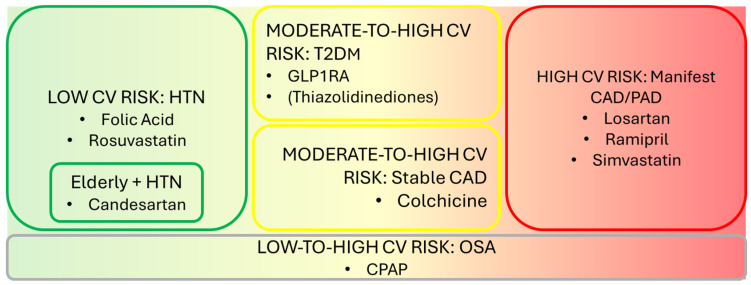
Primary prevention drug strategies according to incremental CV risk.

**Figure 2:**
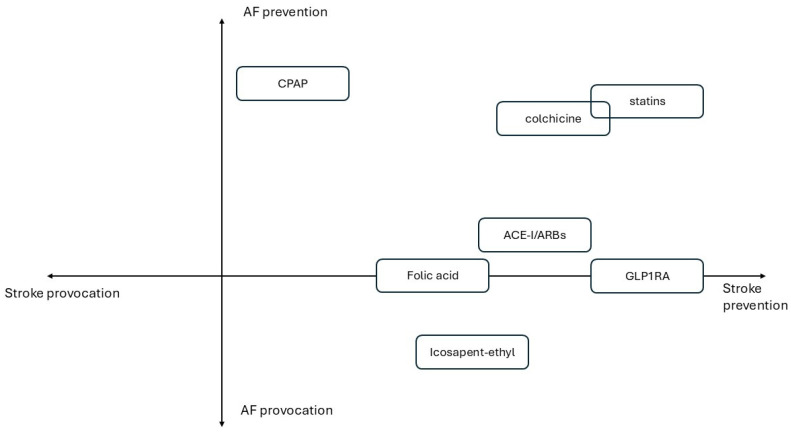
Quadrant-based graph of described treatments based on their effects on AF and stroke onset.

For the purpose of this review, the following are true:

- All RCTs (Table 1) and meta-analyses included (Table 2) are statistically significant for results unless otherwise stated.
- Each drug or drug class or strategy demonstrating stroke-preventative potential has been analyzed in parallel with its potential to prevent AF onset based on the latest meta-analytic evidence.
- The CV risk profile of patients enrolled in RCTs and meta-analyses has been reviewed in order to contextualize drug efficacy according to incremental CV risk.
- Figure 1 illustrates primary prevention drug strategies categorized to incremental CV risk. Figure 2 summarizes treatments based on their effects on AF onset and stroke prevention.

### 3.1. Drugs Mitigating AF Onset and Stroke Risk in Patients at Low CV Risk

Here, apparently healthy patients are referred to as those unaffected by significant vascular conditions and, if any, affected by hypertension, mild dyslipidemia, or CRP increases, as well as recent-onset diabetes.

Folic acid 0.8 mg/die has demonstrated effectiveness in reducing stroke risk in hypertensive patients receiving enalapril, as evidenced by the megatrial CSPPT [199]. The greatest benefit was observed in patients with lower baseline folate levels (<5.6 ng/mL) (2.8% vs. 4.6%, HR 0.61) and, more specifically, in patients with a low platelet count (HR 0.27–0.11 to 0.64) [200].

Rosuvastatin 10 mg/die has been compared with placebo in an RCT called HOPE-3 [201] enrolling more than 12,000 apparently healthy patients, according to the definition described above. The results were consistent with those of the JUPITER trial [23], which recruited apparently healthy patients without CV disease, including non-elevated LDL levels but with increased CRP (>2 mg/L). Concerns have been raised regarding the potential induction of type 2 diabetes mellitus (T2DM) with long-term statin therapy. However, the number needed to treat (NNT) and prevent one stroke compared to the number needed to harm (NNH) and cause a new diabetes diagnosis over a 4-year period is 1/6.2. Moreover, this risk is dose- and time-dependent, meaning it can be modulated accordingly by adjusting statin dosage and the duration of therapy. This favorable ratio supports statin therapy as being beneficial overall despite the potential risk of diabetes development. These findings strongly support rosuvastatin’s role in stroke risk reduction.

Given their benefits, rosuvastatin and folic acid may be regarded as attractive treatment options for apparently healthy patients with nAF who do not qualify for anticoagulation due to a CHA_2_DS_2_-VASc ≤ 1 or subclinical AF detected via wearable technology. This intriguing option should be considered based on the awareness that both atorvastatin and rosuvastatin (according to meta-analytic evidence) [74,75] reduce the onset of AF while keeping in mind that these findings refer to patients at higher CV risk.

The above-mentioned trials enrolled patients aged 60–65 years on average. The SCOPE trial focused on elderly patients (mean age 76 years) with no CVD except for mild-to-moderate hypertension. It found that candesartan administration significantly reduced nonfatal stroke in this elderly population. However, apart from losartan administration in LV hypertrophy and higher risk patients, current evidence does not support any AF-mitigating effect in apparently healthy subjects treated with either ARBs or ACE-Is.

Thus far, in low-CV-risk patients, rosuvastatin and folic acid administration, respectively, in the sixth decade of age and candesartan in the seventh decade of age, could be regarded by physicians as potential stroke-preventative treatments.

### 3.2. Drugs Mitigating AF Onset and Stroke Risk in Patients with Moderate-to-High CV Risk

T2DM diagnosis is a critical shift in the acquisition of further CV risk. It is widely accepted that TZDs and GLP-1RAs are likely to reduce stroke burden [79]. Currently, no data exist regarding the use of these drugs in the non-diabetic population, except for semaglutide in patients with obesity, although no stroke-related outcomes have been reported so far.

Nascent AF very often slips through the net of diabetes, and, once intercepted, it may require therapy adjustments in terms of both anti-ictal strategies and anticoagulation indication. GLP-1RAs are, nowadays, recognized as a safe and effective second-line therapy for T2DM. Two drugs, dulaglutide and semaglutide, held promise to be effective in patients with moderate-to-high and high CV risk, respectively, according to baseline characteristics of registration trials (REWIND [98] and SUSTAIN-6 [100] trials). Since only a small percentage of patients enrolled in these trials had a prior stroke (~10% in REWIND, ~20% in SUSTAIN-6), these drugs have been promoted for primary stroke prevention, particularly in patients with suboptimal glucose control (HbA1c > 7%). Interestingly, semaglutide appears to be the only GLP1-RA that significantly reduces the burden of incident AF [99] whereas dulaglutide has shown a neutral profile [96]. Some concerns exist regarding the potential of GLP-1RAs to exacerbate AF, which warrants further investigation [97].

Stable coronary artery disease (CAD) encompasses a wide spectrum of CV risks, ranging from patients with an Agatstone calcium score of 400 in the absence of any surgical need to those with stable but incomplete coronary artery revascularization. Inflammation plays a major role in the development of both stroke and AF [202]. A low dose of colchicine at 0.5 mg/die has been evaluated in the LoDoCo trial [167], which enrolled more than 5400 patients, ~85% of whom had previously undergone percutaneous coronary intervention (PCI), and randomized them to colchicine or a placebo. Not only did low-dose colchicine show an unexpected stroke incidence reduction over a short follow-up period of 2.4 years, but accumulating meta-analytic evidence suggests that colchicine may also prevent the progression of nascent AF [164]. Notably, low-dose colchicine has a high safety profile, and its efficacy is well established even at low doses. The most common adverse effects are gastrointestinal (GI) disturbances, which are typically mild and can be resolved upon drug discontinuation [164].

These results position colchicine as a potential therapeutic agent not only in reducing stroke risk but also in modulating AF progression, particularly in patients with underlying atherosclerotic inflammatory mechanisms.

### 3.3. Drugs Mitigating AF Onset and Stroke Risk in Patients at High CV Risk

The PAD/CAD patient subset often represents the zenith of the CV risk profile as these individuals frequently have HF and a history of coronary revascularization. In clinical studies, stroke prevention strategies in this population are typically inferred from major adverse cardiovascular event (MACE) deconstruction. ACE-Is and ARBs are among the treatment drugs involved in this pipeline.

The HOPE trial [174] included 9200 high-CV-risk patients randomized to ramipril 10 mg/die or a placebo. The majority of participants had myocardial infarction (MI) and PAD, while only ~10% had a prior stroke, making ramipril an attractive option for primary stroke prevention. While significantly reducing stroke over a short period of a little more than 3 years, ramipril failed to prevent AF, except in patients affected by left ventricular dysfunction (LVD) or hypertrophy. A non-inferiority trial comparing telmisartan (80 mg) to ramipril demonstrated that telmisartan was non-inferior to ramipril for stroke prevention [176].

The LIFE trial [190] randomized more than 9000 patients who were predominantly healthy except for electrocardiogram (EKG)-diagnosed left ventricular hypertrophy (LVH). Approximately 20% suffered from PAD/CAD. They were randomized to losartan or atenolol to assess losartan’s stroke-preventative potential across a timeline of 4.8 years. Patients treated with losartan experienced a 25% lower incidence of stroke compared to the control group [190]. In addition, a post hoc subanalysis showed a substantial reduction in the incidence of AF [191], thus making losartan a suitable choice to prevent stroke and nAF in the presence of EKG-assessed LV hypertrophy. While completing this sequence of incremental CV risk, the HSP trial needs to be mentioned [78].

The HSP trial enrolled patients affected by any arterial occlusive disease, including cerebrovascular disease or diabetes. Patients were randomized to 40 mg of simvastatin or a placebo over a mean timeline of 4.8 years. A subanalysis of patients without prior cerebrovascular disease (those enrolled for a primary prophylaxis intention) showed a significant stroke reduction. However, concerns regarding power analysis arise, as only ~500 patients per group were included in this subanalysis. Tailored strategies mitigating AF onset and stroke risk irrespective of CV risk should be studied.

A significant therapeutic alignment between primary stroke prevention and nascent atrial fibrillation (nAF) prevention lies in obstructive sleep apnea (OSA) treatment with continuous positive airway pressure (CPAP). Indeed, cardiologists can face OSA no matter the CV risk of the patient. As discussed above, the mismatch between evidence coming from RCTs and cohort study data, with the latter solely supporting an anti-ictal effect of CPAP, makes the field currently unclear. The open debate takes into account CPAP compliance, OSA severity, and the extent of comorbidities. On the other hand, there is absolute cause–effect coupling [106] between OSA and AF onset, and a growing amount of findings suggest that CPAP therapy prevents AF onset [121]. Such highly suggestive evidence concerning CPAP potential against stroke has been adopted by the very recent ACC/AHA guidelines for primary stroke prevention [34].

The debate regarding TZD administration has been complicated by the historically harmful effects of rosiglitazone. As a result, TZDs (now primarily represented by pioglitazone) have been relegated to third-line therapy for T2DM. Although associated with a reduction in AF burden [22] in secondary stroke prophylaxis [18,19,20], TZDs should not be recommended in primary prophylaxis. Nonetheless, some striking evidence arising from an impressive Korean registry might suggest rethinking pioglitazone as a promising anti-ictal strategy in pre-diabetic patients and in primary prophylaxis [21]. Specific RCTs addressing this endpoint would be highly valuable and, hopefully, extremely useful.

Lastly, out of the chorus, omega-3 PUFAs are thought to confer only mild protection against coronary heart disease (CHD) and coronary death, whereas stroke outcome remains uncertain [98]. Recently, this uncertainty has been challenged following the positive results of high-dose icosapent ethyl, a pure eicosapentaenoic acid, that reduced MACE and nonfatal stroke [203].

Nonetheless, the REDUCE-IT trial has been questioned because of the unhealthy mineral oil employed in the placebo group, which was likely co-responsible for the impressive beneficial effects registered in the icosapent-ethyl-receiving arm. Moreover, high doses of icosapent ethyl were associated with a higher incidence of AF and peripheral edema, thus strongly diminishing the potential benefits of this nutraceutical compound until further evidence becomes available.

## 4. Conclusions

The incidental detection of “nascent” atrial fibrillation has become increasingly frequent due to the widespread use of wearable technologies and recordings from cardiovascular implantable electronic devices (CIEDs). Once recorded and verified, nascent AF represents a turning point in cardiovascular management, regardless of the patient’s baseline CV risk.

When anticoagulation is indicated, its prescription should be complemented by a shift toward drugs with stroke-preventing properties. This shift becomes even more relevant when anticoagulation indications are unclear or difficult to establish, as noted earlier.

In low-CV-risk patients, folic acid, rosuvastatin, and candesartan in the elderly are safe and effective options when facing the need to mitigate stroke risk. Moreover, statins provide an adjunctive preventive effect against AF occurrence.

Patients with moderate-to-high CV risk almost invariably have diabetes mellitus and/or stable CAD. GLP1RA and particularly dulaglutide and semaglutide have demonstrated (in T2DM-randomized trials) stroke-preventative potential. Evidence regarding AF occurrence ranges from mitigation for semaglutide to neutral effects for dulaglutide but, since some warnings exist in meta-analyses with respect to AF provocation, it is accepted that GLP1RAs are uninfluential.

When treating stable CAD patients, colchicine represents a unique opportunity to mitigate both stroke and AF occurrence risk.

In high-CV-risk patients, who are very often affected by overt or treated arterial disease, ramipril, telmisartan, and losartan (the latter is used in EKG-assessed hypertrophy) promise to reduce stroke while mitigating AF onset in left ventricular dysfunction and/or hypertrophy.

A common underlying factor in AF and stroke is obstructive sleep apnea (OSA). CPAP therapy has demonstrated AF mitigation benefits, and although evidence of its primary stroke prevention effect remains conflicting, it is reasonable to expect a certain degree of prophylactic benefit.

Statin-treated apparently healthy patients should be monitored for potential diabetes onset.

Low-dose colchicine likely appears to be as safe as it is effective for stroke and AF prevention. Patients on treatment with statins and colchicine deserve careful evaluation in terms of the benefit/risk ratio, especially if treated with high doses.

## Figures and Tables

**Table 1:** Randomized clinical trials on stroke-preventative drugs.

Name of Study	Drug	Type of Study	Baseline Features	Risk Reduction (HR—95% CI)
HOPE-3 [14]	Rosuvastatin	Trial—primary prevention	Mild dyslipidemia and HTN	0.53–0.37 to 0.78
JUPITER [23]	Rosuvastatin	Trial—primary prevention	CRP increase (>2 mg/L)	0.52–0.34 to 0.79
CSPPT [199]	Folic acid	Trial—primary prevention	HTN	0.76–0.64 to 0.91
SCOPE [184]	Candesartan	Trial—mainly primary prevention (4% pts affected by stroke at baseline)	Elderly (≥70 yrs) and HTN	0.58—0.33 to 1.00
REWIND [98]	Dulaglutide	Trial—significant primary prevention (20% pts affected by MI/stroke)	T2DM and CV event or CV disease	0.76—0.62 to 0.94
LIFE [190]	Losartan	Trial—mainly primary prevention (4/4% pts affected by stroke/TIA at baseline)	HTN and LVH (20% of CAD/PAD)	0.75—0.63 to 0.89
LoDoCo 2 [167]	Colchicine	Trial—primary prevention	Chronic CAD	0.72—0.57 to 0.92
HOPE [174]	Ramipril	Trial—significant primary prevention (10% pts affected by stroke at baseline)	CAD or PAD or stroke or diabetes + at least one classical CV risk factor	0.68—0.56 to 0.84
SUSTAIN-6 [100]	Semaglutide	Trial—significant primary prevention (10% pts affected by stroke at baseline)	T2DM and CV disease or event + Hba1c > 7% or heart/renal failure	0.61—0.38 to 0.99
HPS [78]	Simvastatin	Trial—primary prevention	PAD or CAD or CVD or diabetes	0.75–0.67 to 0.79

**Table 2:** Meta-analyses on drugs mitigating AF onset.

Author	Drug	Main Clinical Features	No. of Studies	Risk Reduction (HR—95% CI)
Yang et al. [74]	Atorvastatin	Moderate-to-high CV risk	18	0.55–0.38 to 0.81
Liu et al. [75]	Rosuvastatin	Low-to-high CV risk	4	0.70–0.54 to 0.91
Zhang et al. [86]	Pioglitazone	Low-to-high CV risk	7	OR 0.73–0.62 to 0.87
Monami et al. [94]	GLP1-RA	Moderate-to-high CV risk	31	MH-OR 0.87–0.71 to 1.05 *p* = 0.15
Affas et al. [121]	CPAP	Low-to-moderate CV risk	17	OR 0.51–0.27 to 0.97
Tian et al. [164]	Colchicine	Moderate-to-high CV risk	17	0.75–0.68 to 0.83
Healey et al. [178]	ACE-I	Low-to-high CV risk	7	0.72–0.56 to 0.93

## Data Availability

No data were created.

## Abbreviations

ACE-Is	Angiotensin-Converting Enzyme Inhibitors
AC	atrial cardiomyopathy
ACC/AHA	American College of Cardiology/American Heart Association
ACE	Angiotensin-Converting Enzyme
ACE-Is	ACE inhibitors
ACS	acute coronary syndrome
ADMA	asymmetric dimethylated arginine
AF	atrial fibrillation
AHREs	atrial high-rate episodes
Akt	Protein Kinase B
AMPK	AMP-activated Protein Kinase
Ang	angiotensin
ARBs	Angiotensin II Receptor Blockers
AT1	Angiotensin II Type 1 Receptor
AT2	Angiotensin II Type 2 Receptor
BK	bradykinin
CaMKII	Calcium/Calmodulin-dependent Protein Kinase II
CAD	coronary artery disease
CHA_2_DS_2_-VASc	Congestive Heart Failure, Hypertension, Age ≥ 75 (2 points), Diabetes, Stroke/TIA (2 points), Vascular Disease, Age 65–74, Sex Category (Female)
CHARM	Candesartan in Heart failure Assessment of Reduction in Mortality and morbidity
CI	Confidence Interval
CIAFS	Colchicine in Atrial Fibrillation to Prevent Stroke
CIED	Cardiac Implantable Electronic Device
CPAP	continuous positive airway pressure
Hs-CRP	Human soluble C-Reactive Protein
CSPPT	China Stroke Primary Prevention Trial
CV	cardiovascular
DHA	docosahexaenoic acid
EKG	electrocardiogram
eNOS	Endothelial Nitric Oxide Synthase
EPA	eicosapentaenoic acid
ERK	Extracellular Signal-Regulated Kinase
ESC	European Society of Cardiology
ESUS	Embolic Stroke of Undetermined Source
FPP	farnesyl pyrophosphate
GGPP	geranylgeranyl pyrophosphate
GLP-1RA	Glucagon-Like Peptide-1 Receptor Agonist
HbA1c	Hemoglobin A1c
HF	heart failure
HMGB1	High-mobility group protein 1
HMG-CoA	3-Hydroxy-3-Methylglutaryl-Coenzyme A
HOPE	Heart Outcomes Prevention Evaluation
HR	Hazard Ratio
HTN	hypertension
HPS	Heart Protection Study
HSP	Heat Shock Protein
ICD	implantable cardioverter defibrillator
IKK	IκB kinase
IL-1β	Interleukin-1 beta
IL-6	Interleukin-6
IPE	Icosapent ethyl
IRIS	Insulin Resistance Intervention After Stroke
JUPITER	Justification for the Use of Statins in Prevention: An Intervention Trial Evaluating Rosuvastatin
LIFE	Losartan Intervention For Endpoint reduction in hypertension
LoDoCo	Low-Dose Colchicine for Secondary Prevention of Cardiovascular Disease
LVD	left ventricular dysfunction
LVH	left ventricular hypertrophy
MACEs	major adverse cardiovascular events
MAPK	Mitogen-Activated Protein Kinase
MCP-1	monocyte chemoattractant protein-1
MDPI	Multidisciplinary Digital Publishing Institute
MI	myocardial infarction
MICL	Myeloid inhibitory C-type lectin-like receptor
MMPs	matrix metalloproteinases
NADPH Oxidase	Nicotinamide Adenine Dinucleotide Phosphate Oxidase
NF-κB	nuclear factor-kappa B
NLRP3	NLR Family Pyrin Domain-Containing 3
NO	nitric oxide
nAF	nascent atrial fibrillation
ONTARGET	ONgoing Telmisartan Alone and in Combination With Ramipril Global Endpoint
OPG	osteoprotegerin
OSAS	Obstructive Sleep Apnea Syndrome
PAD	peripheral artery disease
P2X7	Purinergic Receptor P2X 7
PFO	Patent Foramen Ovale
PI3K	Phosphoinositide 3-Kinase
PROactive	PROspective pioglitAzone Clinical Trial In macroVascular Events
PROSPER	PROspective Study of Pravastatin in the Elderly at Risk
PUFA	polyunsaturated fatty acid
Rac1	Ras-related C3 botulinum toxin substrate 1
RAAS	renin–angiotensin–aldosterone system
RCT	randomized controlled trial
REDUCE-IT	Reduction in Ischemic Stroke With Icosapent Ethyl
REWIND	Researching cardiovascular Events with a Weekly INcretin in Diabetes
RhoA	Ras Homolog Family Member A
ROCK	Rho-associated kinase
ROS	reactive oxygen species
RR	Relative Risk
SCOPE	Study on COgnition and Prognosis in the Elderly
SELECT	Semaglutide Effects on Heart Disease and Stroke in Patients with Overweight or Obesity
SGLT2-Is	Sodium-Glucose Transporter-2 Inhibitors
SIRT1	Sirtuin 1
SHS	Sleep Heart Study
SPARCL	Stroke Prevention by Aggressive Reduction in Cholesterol Levels
SUSTAIN-6	Semaglutide and Cardiovascular Outcomes in Patients with Type 2 Diabetes
TF	tissue factor
TIA	Transient Ischemic Attack
TGs	triglycerides
T2DM	type 2 diabetes mellitus
TLR4	Toll-Like Receptor 4
TM	thrombomodulin
TNF-α	Tumor Necrosis Factor Alpha
TZDs	thiazolidinediones
